# The Relationship Between Mindfulness and Dental Fear Across Dental Specialties: A Cross-Sectional Study

**DOI:** 10.3390/healthcare14070870

**Published:** 2026-03-28

**Authors:** Gizem Yazdan Özen, Başak Topdağı, Ali Kağan Özen, Nebiha Hilal Bilge, Kübra Aslantaş Akar

**Affiliations:** 1 Department of Orthodontics, Division of Clinical Sciences, Faculty of Dentistry, Kafkas University, Kars 36100, Turkey; dtgzmyazdan@gmail.com; 2 Department of Prosthodontics, Division of Clinical Sciences, Hamidiye Dental Faculty, University of Health Sciences, İstanbul 34668, Turkey; basak.topdagi@sbu.edu.tr; 3 Department of Oral and Maxillofacial Radiology, Division of Clinical Sciences, Faculty of Dentistry, Kafkas University, Kars 36100, Turkey; n.hilalbilge@gmail.com; 4 Department of Endodontics, Division of Clinical Sciences, Faculty of Dentistry, Kafkas University, Kars 36100, Turkey; kubraslantas.3@icloud.com

**Keywords:** dental anxiety, mindfulness, specialties, dental

## Abstract

**Background:** This study aimed to investigate the relationship between dental fear and mindfulness levels, and to examine how this relationship varies across different dental specialties. **Methods:** The Dental Fear Scale (DFS) and the Mindful Attention Awareness Scale (MAAS) were administered to 411 adult patients receiving treatment in six different clinics of the Faculty of Dentistry at Kafkas University. Data were analyzed using the Kruskal–Wallis, Mann–Whitney U, Chi-square, and Spearman’s correlation tests. **Results:** A moderate, significant, and negative correlation was found between MAAS and DFS scores (r = −0.41; *p* < 0.001). Mean scores differed significantly across clinics. Patients in the Prosthodontics Department exhibited the highest levels of fear (62.21 ± 4.62) and the lowest levels of mindfulness (3.22 ± 0.23), whereas patients in the Oral and Maxillofacial Radiology Department demonstrated the lowest fear levels (40.60 ± 15.76) and the highest mindfulness levels (4.30 ± 1.00). Consistent with these score-level differences, the distribution of dental fear categories varied across clinics, with a significantly higher prevalence of high anxiety in the Prosthodontics clinic (75.7%) compared to the Orthodontics and Radiology clinics. **Conclusions:** Higher levels of mindfulness were associated with lower levels of dental fear, and this relationship was consistent across all clinical settings. The study highlights that clinical context significantly influences both mindfulness and fear levels, with invasive specialties showing a higher risk profile. Brief mindfulness-based interventions may serve as effective and feasible strategies to enhance patient cooperation and improve treatment outcomes, particularly in clinics where high levels of fear are prevalent.

## 1. Introduction

Fear is an instinctive and natural response developed by individuals to avoid harm in the face of a known situation [1]. Dental anxiety, on the other hand, arises in the absence of a specific external stimulus and is a psychological condition characterized by intense discomfort and worry associated solely with dental treatment [2,3]. Previous studies have reported that dental anxiety ranks as the fifth most common type of anxiety experienced by individuals [2,3,4]. Dental fear and anxiety can occur at any age, leading to avoidance of dental treatment and consequently exerting a negative impact on oral and dental health [5,6]. Dental anxiety is not merely an isolated fear response, but a multifactorial construct involving cognitive, emotional, behavioral, and physiological components [7,8]. Current evidence indicates that dental anxiety is associated with avoidance of dental care, irregular attendance, poorer oral health outcomes, and reduced oral-health-related quality of life [7,8,9,10]. In addition, psychological and contextual determinants such as previous traumatic experiences, general anxiety/fear, sensory over-responsivity, conditioning processes, vicarious learning, verbal threat, and parental influences play a significant role in the development and maintenance of dental anxiety [8,9]. Taken together, these findings indicate that dental anxiety should be understood within a broader psychosocial framework [7,9]. Within this context, there is a strong theoretical rationale for examining the potential role of protective psychological constructs such as mindfulness in reducing dental fear across different clinical settings.

In the Turkish population, the prevalence of dental fear and anxiety has been reported to range between 21.3% and 23.5%, whereas in other societies this rate varies between 2.5% and 20% [11,12]. This situation contributes to the avoidance of dental services in Türkiye, thereby increasing oral health problems.

Studies in the literature have shown that dental fear and anxiety can differ significantly according to various demographic variables such as age, gender, educational level, socioeconomic status, and previous dental experiences [13,14,15]. Therefore, the accurate assessment of dental fear and anxiety, as well as the identification of individuals who require special approaches in the clinical process, is of critical importance for effective treatment planning [16,17]. In contrast, the concept of mindfulness, which enables the individual to focus on the present experience with attention and non-judgmental acceptance, is considered an important psychological skill in emotional regulation, the reduction in avoidance behaviors, and the development of coping abilities with stress [18,19].

Studies investigating the relationship between mindfulness and dental anxiety have shown that as mindfulness levels increase, dental anxiety decreases at both state and trait levels [20]. Moreover, it has been demonstrated that the reducing effect of mindfulness on dental anxiety is partially mediated by rational thinking, which may contribute to the development of intervention strategies. At the clinical level, mindfulness meditation has been reported to be a feasible and reliable method for managing stress during dental implant procedures, providing beneficial psychological, physiological, and biochemical effects, and even brief interventions have been found to be effective in reducing dental anxiety [21]. In addition, negative associations have been identified between mindfulness and self-compassion with dental anxiety and dental neglect, with dental anxiety playing a partial mediating role in this relationship [22]. Taken together, these findings suggest that mindfulness- and self-compassion-based interventions hold promise for improving oral health behaviors by reducing dental anxiety.

Mindfulness has been increasingly recognized as an important psychological construct influencing anxiety-related processes. Previous research suggests that mindfulness-based interventions operate through several underlying mechanisms, including reductions in rumination and worry, as well as improvements in emotional regulation and non-judgmental acceptance of internal experiences [23]. In particular, the development of mindfulness skills promotes a non-reactive and accepting stance toward thoughts and emotions, thereby reducing repetitive negative thinking patterns and maladaptive cognitive reactivity [23]. Neurobiological evidence further supports these findings, demonstrating that mindfulness practices are associated with changes in brain regions involved in emotion regulation, self-referential processing, and perspective taking [24]. From a psychological perspective, mindfulness-based approaches, including Acceptance and Commitment Therapy, aim to alter individuals’ relationships with internal experiences by fostering acceptance and reducing experiential avoidance [25]. These mechanisms are particularly relevant in the context of dental anxiety, which is a multidimensional construct involving cognitive, emotional, behavioral, and physiological components, and is often associated with avoidance of dental treatment [26]. Therefore, it can be hypothesized that increased mindfulness may reduce dental anxiety by modulating emotional and cognitive processes and decreasing avoidance behaviors.

Despite the growing body of evidence suggesting a negative association between mindfulness and dental anxiety, several important gaps remain in the literature. First, most previous studies have examined this relationship in general populations or single clinical settings, without considering how different dental specialties—characterized by varying levels of invasiveness, treatment duration, and expected pain—may influence both mindfulness and dental fear. Second, the majority of existing research has focused primarily on overall anxiety levels, with limited attention given to how mindfulness interacts with different dimensions or categories of dental fear within diverse clinical contexts. Third, although potential psychological mechanisms such as emotional regulation, cognitive appraisal, and reduction in avoidance behaviors have been proposed, there is a lack of empirical studies integrating these perspectives into comparative clinical designs. Therefore, the present study aims to address these gaps by investigating the relationship between mindfulness and dental fear across multiple dental specialties within the same clinical framework, thereby providing a more context-sensitive understanding of this relationship and offering clinically applicable insights for the development of targeted anxiety management strategies.

In this context, the aim of our study is to evaluate the relationship between dental fear and mindfulness levels in adults and to examine whether this relationship differs according to the clinical discipline to which patients present. Our first hypothesis is that the level of mindfulness is inversely proportional to the level of fear, and our second hypothesis is that this proportionality will differ across clinical specialties. To test these hypotheses, dental fear will be measured with the Dental Fear Scale (DFS) [6,27] and mindfulness with the Mindful Attention Awareness Scale (MAAS) [18]; the findings obtained are expected to contribute to the development of individualized anxiety management in clinical processes and to the structuring of brief, feasible mindfulness-based interventions.

## 2. Methods

This study was a single-center, multi-clinic, descriptive, and analytical cross-sectional research. Data were collected between June and September 2025 from individuals attending the clinics of Orthodontics, Endodontics, Prosthodontics, Oral and Maxillofacial Surgery, Periodontology, and Oral and Maxillofacial Radiology at the Faculty of Dentistry, Kafkas University. Clinical trial number: not applicable.

Ethical approval for this study was obtained from the Ethics Committee of the Faculty of Medicine, Kafkas University, on 24 June 2025 (Approval No: KAÜ-TFEK 2025/06/10). Written informed consent was obtained from all participants. The study was conducted in accordance with the principles of the Declaration of Helsinki.

The study population consisted of adult patients aged 18 years and older who were applying to the relevant clinics for the first time for examination or treatment. Participants who presented with emergency complaints were included in the study; however, no distinction was made between emergency and routine patients during the analysis phase, and all participants were evaluated as a single group within their respective specialties. Inclusion criteria were being 18 years of age or older, possessing the cognitive capacity to complete the questionnaires independently, and providing written informed consent. Exclusion criteria included having a pre-existing psychiatric disorder, current use of psychiatric or psychotropic medication, prior experience with professional mindfulness-based therapy or training and providing incomplete or inconsistent responses to the questionnaires. Regarding the clinical settings, the Department of Pedodontics was excluded as the study specifically targeted the adult population. Furthermore, the Department of Restorative Dentistry was not included due to logistical constraints and a lack of field researchers available to administer the surveys. This selection was made to maintain a standardized data collection process and ensure consistency in the administration of the questionnaires.

A structured questionnaire consisting of two parts was used as the data collection tool. The Turkish version of the DFS, which has been demonstrated to be a reliable and valid tool to assess dental fear levels in various Turkish populations, was utilized in this study [6,11,27,28,29,30,31]. In the first part, the Dental Fear Scale (DFS) [6,27,31] was applied to assess dental fear levels. The DFS is a 20-item, 5-point Likert-type scale primarily evaluating reactions to dental stimuli such as the sight of the anesthetic needle and the sound or sensation of the drill. Higher total scores indicate higher levels of dental fear, with the total score obtained by summing the responses to all items. According to DFS scores, participants were classified into four groups: very high fear (Group 1: >80 points), high fear (Group 2: 60–80 points), moderate fear (Group 3: 40–60 points), and low fear (Group 4: <40 points). In the second part of the study, the Mindful Attention Awareness Scale (MAAS) [18] was utilized to measure mindfulness levels, an instrument that has been widely validated across diverse Turkish populations, demonstrating consistent single-factor structures and high internal reliability in various recent studies [32,33]. This scale, consisting of 15 items with a 6-point Likert structure, evaluates individuals’ awareness and attention to the present moment rather than acting on ‘automatic pilot’. The instrument has been validated for the Turkish population, demonstrating a consistent single-factor structure where higher scores indicate greater mindfulness.

During the data collection process, participants were informed in the clinical waiting area before treatment and completed the questionnaires face-to-face. Confidentiality of participant identities was ensured, and data were analyzed using coded identifiers only.

To ensure the representativeness of the sample for a broader patient population, a balanced distribution was maintained across six different dental specialties through a simple randomization process. The sample size was calculated using G*Power 3.1.9.7 software. Based on a one-way ANOVA with a medium effect size (f = 0.25), a significance level of α = 0.05, and a statistical power of 0.80 (1 − β), the analysis indicated that a minimum of 67 participants per group was required. As a result, the minimum required number of participants was reached in nearly all groups, achieving a total sample size of 411 participants and providing sufficient statistical power to generalize the findings.

Data analysis was performed using SPSS version 25.0. Descriptive statistics were calculated, and data distribution was evaluated using the Shapiro–Wilk test. The Kruskal–Wallis test was used to show the inter-clinic distribution of DFS and MAAS scores that did not show a normal distribution. Inter-clinic pairwise comparisons were analyzed using the Mann–Whitney U test. The association between DFS classification and clinical groups was examined using the Chi-square test. Relationships between DFS and MAAS scores were evaluated with Spearman correlation analysis. A *p*-value of 0.05 was considered the threshold for statistical significance at a %95 confidence interval.

## 3. Results

A total of 411 participants were included in the study, consisting of 276 females (67.15%) and 135 males (32.85%) (Table 1). The mean age was 27.55 ± 3.56 years for females and 29.51 ± 2.15 years for males.

According to the Kruskal–Wallis test, both MAAS and DFS scores showed significant differences among the clinics (*p* < 0.001). The distribution of DFS and MAAS scores by clinics is presented in Table 2.

Pairwise comparisons analyses revealed that patients attending the Prosthodontics (PDT) clinic had significantly higher DFS scores compared to all other clinics. Patients in the Orthodontics and Oral and Maxillofacial Radiology (OMFR) clinics had significantly lower DFS scores compared to those in the Endodontics, Oral and Maxillofacial Surgery (OMFS), and Prosthodontics clinics. Additionally, patients in the OMFS clinic had significantly higher DFS scores than those in the Periodontology clinic (*p* < 0.05).

Regarding MAAS scores, patients in the Prosthodontics clinic had significantly lower scores compared to all other clinics. Conversely, patients in the Oral and Maxillofacial Radiology clinic had significantly higher MAAS scores compared to those in the Prosthodontics, Endodontics, Periodontology and Oral and Maxillofacial Surgery clinics (*p* ≤ 0.05). Patients in the Orthodontics had significantly higher MAAS scores compared to those in the Prosthodontics, Endodontics, and Oral and Maxillofacial Surgery clinics (*p* ≤ 0.05).

A significant negative correlation was found between MAAS and DFS scores (r = −0.41, *p* < 0.001).

The Cronbach’s alpha internal consistency coefficient calculated for reliability was 0.92 for the DFS and 0.89 for the MAAS.

There was a significant association between DFS categorization and the clinics. The distribution of DFS categories across the clinics is presented in Table 3. While low and moderate anxiety levels were high in patients presenting to the Oral and Maxillofacial Radiology, Orthodontics, and Endodontics clinics, high anxiety levels were significantly higher in the Prosthodontics.

**Figure 1:**
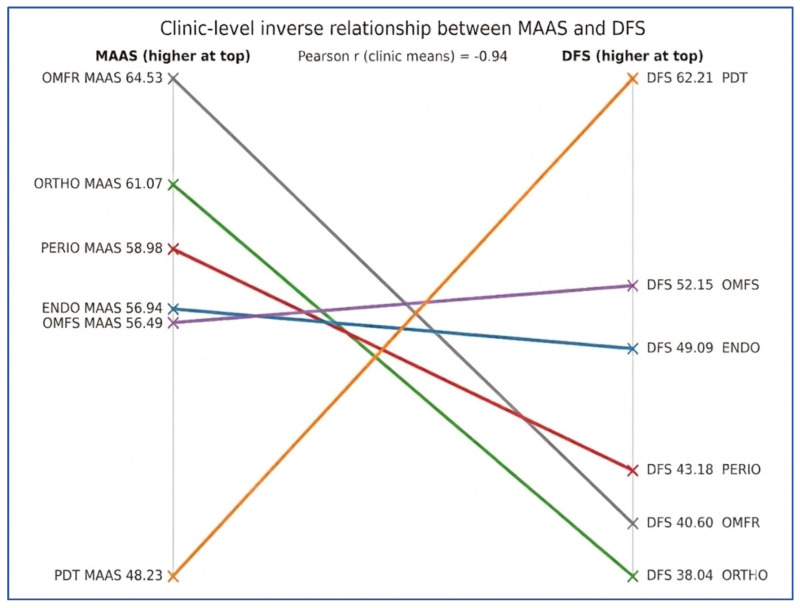
Scatter plot illustrating the inverse correlation between Mindful Attention Awareness Scale (MAAS) and Dental Fear Scale (DFS) scores across the study population. OMFR: Oral and Maxillofacial Radiology; ORTHO: Orthodontics; PERIO: Periodontology; ENDO: Endodontics; OMFS: Oral and Maxillofacial Surgery; PDT: Prosthodontics.

A significant, moderate, negative correlation was observed between mindfulness and dental fear across the entire sample (Spearman’s ρ = −0.41; *p* < 0.001). Accordingly, higher mindfulness scores were associated with lower levels of dental fear, and this inverse relationship is illustrated in Figure 1. However, this trend was not equally evident across all departments. Notably, in clinically demanding specialties involving invasive procedures, such as Endodontics and Oral and Maxillofacial Surgery, relatively higher mindfulness scores were accompanied by persistently elevated fear levels.

## 4. Discussion

In this study, two main hypotheses were tested. The first hypothesis was that there would be a significant relationship between mindfulness level and dental fear. Our findings showed a significant and negative correlation between MAAS and DFS, indicating that higher levels of mindfulness are associated with lower levels of dental fear, thereby confirming our first hypothesis. The second hypothesis—that the mindfulness-fear relationship varies by clinical discipline—was also supported. Patients in Prosthodontics (PDT) reported the highest fear and lowest mindfulness, while Oral and Maxillofacial Radiology (OMFR) patients exhibited the opposite profile. Furthermore, this clinical diversity was clearly reflected in the distribution of dental fear categories, where high-risk and low-risk clinics exhibited distinct and consistent patterns (Table 3), thereby providing comprehensive support for our second hypothesis at both the score and categorical levels.

This study demonstrated the relationship between mindfulness (MAAS) and dental fear (DFS) across a large sample (*n* = 411). First, a moderate and significant negative correlation was detected between MAAS and DFS (r = −0.41; *p* < 0.001). This finding is consistent with the theoretical and empirical literature describing the role of mindfulness in the regulation of anxiety and fear [18,19,20]. On the other hand, mean levels differed significantly across clinics: Prosthodontics (PDT) patients had the highest DFS (62.21 ± 4.62) and lowest MAAS (3.22 ± 0.23) profile, while Oral and Maxillofacial Radiology (OMFR) patients had the lowest DFS (40.6 ± 15.76) and highest MAAS (4.30 ± 1.00). In Orthodontics, fear levels were even lower (DFS = 38.04 ± 14.03). Consistent with these score-level differences, the categorical distribution in Table 3 revealed that 75.7% of PDT patients exhibited high anxiety, while the proportions of low fear were 53.8% in OMFR and 60.9% in Orthodontics, demonstrating the influence of clinical context—such as invasiveness and pain expectation—on patient psychology.

The inter-clinic pattern is consistent with the nature of treatment and the patient journey. PDT patients often undergo multi-stage, lengthy, and frequently invasive procedures; when combined with previous negative experiences and a tendency for delayed attendance, high fear levels are expected [34]. Conversely, the significantly lower fear and higher mindfulness profile observed in the OMFR clinic likely reflect the predominantly non-invasive and diagnostic nature of radiological procedures. Radiological examinations are typically brief and lack primary anxiety triggers such as needles or high-speed drills. These observed differences may reflect the inherent nature of clinical procedures rather than pre-existing psychological variations. For instance, higher fear in Prosthodontics and Endodontics is potentially associated with the expectation of prolonged drilling and complex local anesthetic injections, whereas the low fear profile in Radiology is associated with the brief, touch-free, and non-sensory nature of extraoral imaging. Similarly, the predictability and long-term, non-surgical focus of Orthodontic adjustments contribute to lower anxiety levels compared to the acute stress of Oral Surgery. The literature indicates that invasive interventions trigger higher fear, while anxiety is reported less in specialties that are relatively more ‘predictable’ and involve lower pain expectations, such as orthodontics [9,35]. Our data quantitatively support this pattern, suggesting that the clinical context and the specific demands of the procedure are the dominant factors shaping the patient’s psychological state.

Our finding of a negative correlation (r = −0.41) aligns with the broader psychological framework of Emotion Regulation Theory. In the context of dentistry, mindfulness may act as a ‘cognitive buffer’. Individuals with higher MAAS scores are likely to perceive dental stimuli (e.g., the sound of the drill or the sensation of an injection) as transient sensory experiences rather than catastrophic threats. This is consistent with behavioral studies suggesting that mindfulness promotes decentering, allowing patients to observe their anxiety without becoming overwhelmed by it [20,23]. This psychological flexibility explains why the inverse relationship remained consistent across all specialties, regardless of the procedure’s invasiveness.

With regard to the mindfulness–fear relationship, our correlation coefficient (r = −0.41) falls within the same range as those reported by Brown and Ryan [18] in general anxiety contexts and recent correlation coefficients reported in the dental context, thus providing both statistical and clinical significance [20,34]. Yao et al. reported that in mindful individuals, the “acting with awareness” component adds value, explaining the relationship through more rational evaluation and less catastrophizing; the trend in our study is consistent with this mechanism [20,36]. In the present study, higher MAAS scores were consistently associated with lower DFS scores across all clinics. Furthermore, our large sample size and multi-clinic design are valuable in suggesting that the observed association may be a consistent phenomenon independent of clinical context.

For dental practitioners, these results suggest that ‘chair-side’ anxiety management should be stratified based on the clinical branch. In high-fear departments like Prosthodontics, practitioners could implement ‘Micro-Mindfulness’ protocols [21,37]. These involve brief (2–3 min) diaphragmatic breathing, which is theorized to stimulate the vagus nerve and counteract the ‘fight-or-flight’ response, or sensory grounding exercises such as the 5–4–3–2–1 technique to redirect the patient’s focus from internal catastrophic thoughts to the present environment [7,38]. By utilizing these sensory anchors, practitioners may help modulate the neural pathways associated with dental phobia. Furthermore, encouraging a non-judgmental awareness of physical sensations promotes decentering, allowing patients to perceive stimuli like the sound of the drill or the pressure of an injection as transient experiences rather than overwhelming threats. This psychological shift is crucial for breaking the ‘avoidance-pain’ cycle frequently seen in invasive specialties, where treatment delays lead to more complex and painful procedures [34]. Successfully managing a single appointment through these brief interventions can deconstruct the patient’s ‘dentist-equals-pain’ schema and improve long-term attendance. Ultimately, acknowledging the patient’s anxiety and integrating these low-cost, feasible mindfulness modules can significantly enhance patient cooperation, reduce perceived pain, and improve overall treatment outcomes.

Strengths of our study include a large and heterogeneous sample from six different clinics, the use of valid and reliable scales (DFS, MAAS), and the application of appropriate non-parametric tests. Despite its contributions, several limitations of this study must be explicitly acknowledged. First, the cross-sectional design precludes causal inferences; thus, associations between mindfulness and dental fear should be interpreted without implying temporal precedence. Second, although the total sample size (N = 411) was statistically sufficient, the study was conducted in a single university center, which may introduce sampling bias and limit the generalizability of the findings to different sociocultural contexts. Third, the reliance on self-reported measures (DFS and MAAS) carries an inherent risk of social desirability bias or subjective interpretation, which may not fully capture the physiological or behavioral dimensions of dental anxiety. Furthermore, while the majority of dental specialties were represented, the exclusion of pedodontics and restorative dentistry due to logistical constraints represents a further limitation that should be addressed in multi-center future research.

In addition, potential confounding factors such as age, gender, socioeconomic status (SES), and previous traumatic dental experiences were not controlled for in the primary analysis. This methodological choice aimed to highlight the direct association between mindfulness and dental fear across clinical disciplines, without the potentially confounding effects of demographic variables. By focusing on bivariate associations, we aimed to highlight the ‘raw’ clinical profiles of the branches and the fundamental role of mindfulness without the noise of multiple co-variates. However, we acknowledge that the absence of multivariable regression or ANCOVA models to adjust for these confounders represents a limitation. Future research should test clinical differences while adjusting for age, gender, SES, and previous experiences using multivariate modeling, and examine potential mediators (catastrophizing, pain expectation, intolerance of uncertainty) and moderators (general anxiety, health behavior profiles) to provide even more robust insights into the independent effect of mindfulness.

In conclusion, mindfulness level is significantly and consistently correlated with lower dental fear; although mean levels differ across clinical contexts, the direction of the relationship does not change, and severe fear cases are observed in every clinic. These data suggest that anxiety management in dentistry is not solely the responsibility of certain specialties but should be considered a fundamental clinical competency across all disciplines. The systematic integration of mindfulness-based brief interventions and patient-centered communication offers an effective and feasible strategy to break the cycle of avoidance, promote routine care, and improve treatment outcomes. This study provides directly translatable recommendations for clinical practice and establishes a strong foundation for multicenter, prospective, and intervention-focused research.

## 5. Conclusions

This study examined the relationship between mindfulness levels and dental fear, as well as how this relationship varies across different dental clinical disciplines. The findings indicated that

- There is a significant negative correlation between mindfulness and dental fear (r = −0.41; *p* < 0.001).
- Mean values and clinical profiles differed significantly across clinics; Prosthodontics patients exhibited the highest dental fear and the lowest mindfulness, whereas Radiology and Orthodontics patients showed a lower fear profile.
- The distribution of fear categories reflected these clinical differences, with a high prevalence of severe anxiety in invasive specialties, emphasizing the need for department-specific anxiety management.

These results suggest that mindfulness is a significant associated factor in the management of dental anxiety and should be considered across all clinical disciplines. Particularly in high-risk clinical contexts, the integration of brief mindfulness practices into pre-treatment routines may enhance patient cooperation and improve the overall treatment experience.

## Figures and Tables

**Table 1:** Distribution of the study population across different dental specialties.

Dental Specialty (Department)	*n*	Percentage (%)
OMFR ^1^	78	19%
PDT ^2^	70	17%
Endodontics	69	16.8%
Periodontology	57	13.9%
OMFS ^3^	68	16.5%
Orthodontics	69	16.8%
Total	411	100.0%

^1^ Oral and Maxillofacial Radiology, ^2^ Prosthodontics, ^3^ Oral and Maxillofacial Surgery.

**Table 2:** Distribution of MAAS and DFS scores across dental clinics.

	MAAS		DFS	
	Mean ± Std	Median (q1–q3)	Mean ± Std	Median (q1–q3)
OMFR ^1^	64.53 ± 15.1	64.50 (55–77.25)	40.60 ± 15.76	35.50 (28–53.25)
PDT ^2^	48.23 ± 3.47	48 (45–51)	62.21 ± 4.62	63 (59.75–65.25)
Endodontics	56.94 ± 13.62	54 (47–67.50)	49.09 ± 16.25	50 (37–60.50)
Periodontology	58.98 ± 17.17	57 (49–72)	43.18 ± 17.49	42 (26.5–53)
OMFS ^3^	56.49 ± 13.75	52 (46–66.75)	52.15 ± 16.34	56 (45.25–63)
Orthodontics	61.07 ± 12.62	61 (51.50–72)	38.04 ± 14.03	34 (27–45)

^1^ Oral and Maxillofacial Radiology, ^2^ Prosthodontics, ^3^ Oral and Maxillofacial Surgery.

**Table 3:** Distribution of DFS categories across dental clinics.

	Low Anxiety		Moderate Anxiety		High Anxiety		Very High Anxiety	
	*n*	%	*n*	%	*n*	%	*n*	%
OMFR ^1^	42	53.8	28	35.9	7	9	1	1.3
PDT ^2^	0	0	17	24.3	53	75.7	0	0
Endodontics	19	27.5	31	44.9	17	24.6	2	2.9
Periodontology	26	45.6	20	35.1	9	15.8	2	3.5
OMFS ^3^	13	19.1	26	38.2	27	39.7	2	2.9
Orthodontics	42	60,9	22	31.9	4	5.8	1	1.4
Total	142	34.5	144	35	117	28.5	8	1.9

^1^ Oral and Maxillofacial Radiology, ^2^ Prosthodontics, ^3^ Oral and Maxillofacial Surgery.

## Data Availability

Data supporting the findings of this article may be shared upon reasonable request and with the approval of all authors.

## Abbreviations

The following abbreviations are used in this manuscript:

DFS	Dental Fear Scale
MAAS	Mindful Attention Awareness Scale
OMFR	Oral and Maxillofacial Radiology
PDT	Prosthodontics
OMFS	Oral and Maxillofacial Surgery
CBT	Cognitive Behavioral Therapy
MBCT	Mindfulness-Based Cognitive Therapy
MBSR	Mindfulness-Based Stress Reduction
SES	Socioeconomic Status
ANOVA	Analysis of Variance
ANCOVA	Analysis of Covariance
SPSS	Statistical Package for the Social Sciences
